# Differential RNA aptamer affinity profiling on plasma as a potential diagnostic tool for bladder cancer

**DOI:** 10.1093/narcan/zcac025

**Published:** 2022-08-22

**Authors:** Søren Fjelstrup, Daniel M Dupont, Claus Bus, Jan J Enghild, Jørgen B Jensen, Karin Birkenkamp-Demtröder, Lars Dyrskjøt, Jørgen Kjems

**Affiliations:** Interdisciplinary Nanoscience Center (iNANO), Aarhus University, Aarhus, Denmark; Interdisciplinary Nanoscience Center (iNANO), Aarhus University, Aarhus, Denmark; Interdisciplinary Nanoscience Center (iNANO), Aarhus University, Aarhus, Denmark; Department of Molecular Biology and Genetics (MBG), Aarhus University, Aarhus, Denmark; Department of Urology, Aarhus University Hospital, Aarhus N, Denmark; Department of Clinical medicine, Aarhus University, Aarhus, Denmark; Department of Molecular Medicine, Aarhus University Hospital, Aarhus, Denmark; Department of Clinical medicine, Aarhus University, Aarhus, Denmark; Department of Molecular Medicine, Aarhus University Hospital, Aarhus, Denmark; Department of Clinical medicine, Aarhus University, Aarhus, Denmark; Interdisciplinary Nanoscience Center (iNANO), Aarhus University, Aarhus, Denmark; Department of Molecular Biology and Genetics (MBG), Aarhus University, Aarhus, Denmark

## Abstract

The molecular composition of blood is a signature of human health, reflected in the thousands of blood biomarkers known for human diseases. However, establishing robust disease markers is challenging due to the diversity of individual samples. New sequencing methods have simplified biomarker discovery for circulating DNA and RNA while protein profiling is still laborious and costly. To harness the power of high-throughput sequencing to profile the protein content of a biological sample, we developed a method termed APTASHAPE that uses oligonucleotide aptamers to recognize proteins in complex biofluids. We selected a large pool of 2′Fluoro protected RNA sequences to recognize proteins in human plasma and identified a set of 33 cancer-specific aptamers. Differential enrichment of these aptamers after selection against 1 μl of plasma from individual patients allowed us to differentiate between healthy controls and bladder cancer-diagnosed patients (91% accuracy) and between early non-invasive tumors and late stage tumors (83% accuracy). Affinity purification and mass spectrometry of proteins bound to the predictive aptamers showed the main target proteins to be C4b-binding protein, Complement C3, Fibrinogen, Complement factor H and IgG. The APTASHAPE method thus provides a general, automated and highly sensitive platform for discovering potential new disease biomarkers.

## INTRODUCTION

The physiology, life-style and health state of an individual are reflected in the composition of the blood, as illustrated by the numerous disease biomarkers reported in plasma. In particular, the profiling of genetic material (RNA and DNA) has advanced rapidly in recent years due to the advent of next generation sequencing (NGS), which has led to the discovery of a wide range of circulating RNA and DNA biomarkers with strong predictive values for diseases such as cancer (1,2). Also, many protein biomarkers have been identified in blood using mass spectrometry but despite its high sensitivity, this approach has much lower throughput and remains relatively costly (3).

In this study, we developed a novel RNA aptamer-based strategy termed APTASHAPE that allows high throughput profiling of global protein composition in any biofluid using NGS as a read-out. The method utilizes the capacity of chemically modified, serum-stable RNA oligonucleotides to form sequence-dependent functional shapes (RNA aptamers) that can specifically interact with protein epitopes (4). The RNA oligonucleotides are made serum-stable by the introduction of a 2′-Fluoro (2′F) modification on the ribose sugar of the pyrimidines. This modification protects the RNA from autohydrolysis and degradation by RNases (5,6) The aptamers are selected from very large (10^15^) randomized 2′F-pyrimidine-modified RNA libraries by iterative selection for binding to all components in plasma by systematic evolution of ligands by exponential enrichment (SELEX; (7,8)). Previous studies have shown that specific pools of aptamers can be generated for complex targets such as exosomes (9), cells (10) and tissue samples (11), but selections have traditionally been conducted to an extent where the final pool of sequences shows very little diversity.

Implementation of deep NGS methods to analyze aptamer sequences has enabled much more comprehensive analysis of highly diverse aptamer pools. We have previously demonstrated that highly diverse RNA aptamer libraries, selected towards a single protein, can be used to map single amino acid substitutions and interaction sites for binding partners (12). In the present study, we have taken a similar approach but directed the selection towards the full complexity of human plasma, constituting a mixture of >10 000 proteins (13).

As a proof of concept, we tested the APTASHAPE method on plasma samples from bladder cancer patients. The 5-year survival rate for bladder cancer patients depends on the stage of diagnosis: 95% for non-muscle invasive (Ta) bladder cancer, declining to 69% for muscle invasive (T2–T4) and to 5% if distant metastasis is diagnosed (https://www.cancer.org/research/cancer-facts-statistics/all-cancer-facts-figures/cancer-facts-figures-2019.html, https://www.cancer.net/cancer-types/bladder-cancer/statistics) Hence, a key to successful treatment is accurate cancer staging and associated optimal treatment but to date, only three FDA-approved tests for bladder cancer are available for biofluids and none of them are currently recommended for diagnosis, partly due to the poor sensitivity for low grade tumors (16%-47%) (https://www.cancer.org/research/cancer-facts-statistics/all-cancer-facts-figures/cancer-facts-figures-2019.html, https://www.cancer.net/cancer-types/bladder-cancer/statistics). This means that non-invasive methods for the accurate detection of bladder cancer development are currently lacking.

Here, we demonstrate that the 1000 most abundant aptamers obtained from an APTASHAPE training set can distinguish Ta and T2–T4-stage bladder cancer patients from individuals without diagnosed bladder cancer with >90% accuracy. Among these 1000, the 33 aptamers with the highest capacity to distinguish patients with bladder cancer from controls, or early stage cancer from late stage cancer, were produced as individual clones and used to pull down their cognate target proteins. Mass spectrometry analysis identified a number of previously identified bladder cancer biomarkers, highlighting the potential of the method to identify bladder cancer patients based on plasma samples.

## MATERIALS AND METHODS

### Patient sampless

Plasma samples were collected at Aarhus University Hospital, Skejby between 2008 and 2013. Samples were obtained from patients diagnosed with bladder cancer, median age 69 or from a control group from patients without cancer in the bladder or urinary tract. Samples from both groups were age-matched.

The data consists of two cohorts. A training cohort with plasma from 32 control individuals, 32 patients diagnosed with Ta bladder tumors and 32 patients diagnosed with T2–T4 cancer and a validation cohort of eight patients with bladder tumor stage Ta, four with bladder cancer stage T2–T4, and 10 control samples, which were a subset of the samples used in the training set. For all sets of samples, the patients were age-matched and the samples were collected at the same facilities. Informed written consent to take part in future research projects was obtained from all patients, and the specific project was approved by the National Committee on Health Research Ethics (#1706291 and #1708266).

### Collection of blood

10 mL EDTA blood (Vacutainer with K2EDTA) was drawn from the patient. The container was inverted 8–10 times to mix blood with the anticoagulant. The mixture was centrifuged 10 min at 3000 g at room temperature and the plasma supernatant was pipetted into 4.5 ml TPP cryotubes (TPP Techno Plastic Products AG). Tubes with plasma samples were frozen and stored immediately at –80°C.

### SELEX protocol

The 2′F-pyrimidine-modified RNA pool was prepared as described previously (4) with minor modifications. Briefly, dsDNA was prepared by primer annealing and Klenow DNA extension (Thermo Fisher Scientific) using the oligonucleotides 5′-CGCGGATCCTAATACGACTCACTATAGGGGCCACCAACGACATT-3′ (forward oligo) and 5′-GATCCATGGGCACTATTTATATCAAC-N36-AATGTCGTTGGTGGCCC-3′ (pool oligo) obtained from Integrated DNA Technologies. dsDNA was purified by 6% non-denaturing PAGE (National Diagnostics) and transcribed in a reaction containing 80 mM HEPES (pH 7.5), 30 mM DTT, 25 mM MgCl_2_, 2 mM spermidine–HCl, 2.5 mM of ATP and GTP (Thermo Fisher Scientific), 2.5 mM of 2′F-dCTP and 2′F-dUTP (MetkinenChemistry), 100 μg/ml BSA Thermo Fisher Scientific), 0.5 μM dsDNA template and 150 μg/ml mutant T7 RNA polymerase Y639F. The dsDNA was then digested by DNA polymerase I (Thermo Fisher Scientific) and the 2′F-pyrimidine RNA pool purified by denaturing urea–PAGE.

To select aptamer pools for plasma proteins, 1 μl of EDTA-plasma was conjugated to 1 mg of NHS-activated magnetic beads (Thermo Fisher Scientific) overnight in HBS (20 mM HEPES pH 7.4, 140 mM NaCl) at 4°C. Plasma derived from patients diagnosed with T2–T4 stage bladder cancer was applied for positive selection. For counter-selection, magnetic beads were prepared with plasma protein material obtained from bladder cancer negative patients. Beads were washed using SELEX wash buffer (SWB; HBS including 2 mM MgCl_2_, 2 mM CaCl_2_, 5 mM KCl and 0.01% Tween20) and remaining reactive sites blocked with SWB supplemented with 0.1% BSA for 1 h. For the first round of selection, the 2′F-pyrimidine-modified RNA pool (5 copies of 10^15^ different sequences or 8.5 nmol) was refolded by heating to 95°C for 2 min and cooling on ice and counter-selection performed for 1 hour using SELEX buffer (SB; SWB supplemented with 100 ug/ml tRNA and 0.1% BSA). The supernatant from the counter-selection was subsequently transferred to positive selection magnetic beads and binding allowed for 1 hour (selection). Beads were then washed 3 times with SWB and Superscript III reverse transcription (Thermo Fisher Scientific) performed directly on the washed beads using the reverse oligo 5-CCCGACACCCGCGGATCCATGGGCACTATTTATATCAA-3′ (IDT). cDNA was converted to dsDNA using the Thermo Scientific Phusion High-Fidelity DNA Polymerase PCR system with forward and reverse oligos, and the optimal number of PCR cycles determined from a small-scale reaction prior to the full-scale reaction to reduce PCR bias. dsDNA was then digested by BamHI (Thermo Fisher Scientific), purified by the GeneJet PCR purification Kit (Thermo Fisher Scientific), and transcribed to produce RNA for the following round of selection. For subsequent rounds of selection, 200 pmol of RNA pool was applied.

The RNA pools after four rounds of selection were used for branched selection (i.e. one round of parallel selection for each patient sample). The branched selections were performed as described for the aptamer pool selections, however without a counter selection step. For each PCR sample, forward and reverse oligos containing specific barcode sequences were used to allow sample multiplexing during next-generation sequencing. Barcoded PCR samples were submitted to the Beijing Genomics Institute (BGI) for PCR-free pool preparation and one-lane 2 × 100 (Paired End) Illumina HiSeq 4000 sequencing. Reads were subsequently subjected to demultiplexing, pair-mate joining, de-replication and clustering as described previously (12) producing a table of the sequences and the number of times each sequence was observed. Only sequences observed at least 4 times were included. Sequence copy numbers were divided by the total read number for each sample to estimate sequence frequency as the fraction of pool in percent allowing comparison of values across samples.

### Aptamer discovery Scripts

A linear regression model was built for the training set using unweighted models and with no confounding factors using the function lm from base R (14). The *P*-values were adjusted using the Benjamini–Hochberg method, using the p.adjust function from base R (14), a false discovery rate of 0.01% and a 0.5 change in regression coefficient between healthy and cancer samples and a 0.25 regression coefficient increase or decrease was used between the early and late stage cancer samples. The aptamers found to be significant were drawn in a variable clustered heatmap using the pheatmap (15) package. PCA analysis was performed using the prcomp function from base R (14) and visualized using the ggplot2 package (16). The datawrangling was performed using the ReShape (17), SummarizedExperiment (18), and TidyVerse (19) packages. Calculation of Damerau–Levenshtein distance, used for merging sequences stemming from single nucleotide differences, was done with the vwr (20) package. Experimental scripts are available upon request, all analyses were written and run using RStudio ServerVersion 1.3.1073 (21) in R version 4.0.2

### Protein pull-down and SDS-PAGE analysis

Biotinylation of aptamers was performed by 3′-end ribose oxidation using sodium-metaperiodate followed by reaction with EZ-link Biotin-LC-Hydrazide using the standard protocol of the provider (Thermo Fisher Scientific). 400 pmol of biotinylated aptamer was captured on 100 μl of streptavidin magnetic beads (Thermo Fisher Scientific) in HBS buffer for 30 min. Beads were washed 3 times with 500 μl SWB and incubated with SB buffer supplemented with 10% plasma sample for 30 minutes. Beads were then washed 3 times with 500 μl SWB and protein material eluted with HBS 50 mM EDTA. Eluates were analyzed by SDS-PAGE using NuPAGE 4–12% Bis–Tris Gels (Thermo Scientific).

### Mass spectrometry

Mass spectrometry-based protein identification. Bands of interest were excised from Coomassie blue stained SDS-PAGE gels, cut in small cubes, and washed in water. The gel pieces were then incubated in acetonitrile and rehydrated in 0.1 M ammonium bicarbonate. Finally, the gel pieces were swelled in 50 mM ammonium bicarbonate containing 25 mg/ml trypsin before ammonium bicarbonate was added to cover the pieces. The samples were digested for approximately 16 h at 37°C. Following digestion, the tryptic peptides were purified on C18 stage tips. LC–MS was performed using online reverse-phase separation of peptides over a 15 cm column packed in-house with Reprosil-Pur 120 C18-AQ 3 μM resin (Dr. Maisch GmbH) on an EASY-nLC 1200 (Thermo Fisher Scientific) and an Orbitrap Eclipse Tribrid mass spectrometer (Thermo Fisher Scientific) running in data-dependent acquisition mode. For protein identification MS files were converted to the generic Mascot format (MGF) using Raw converter (version 1.1.0.18, Scripps Research Institute) (22), and the generated peak lists were searched against the human Swiss-Prot database (23) using the Mascot search engine (Matrix Science) and the following search parameters: MS tolerance of 10 ppm, MS/MS tolerance of 0.1 Da, trypsin digestion with one missed cleavages, carbamidomethyl as a fixed modification, and oxidized methionine as a variable modification. The main proteins bound were defined as the most abundant protein fragments identified in the MS. With a score of at least 500 and at least twice the score of the highest score of keratin. Keratin is known as a common contaminant, and it was therefore used as the baseline for measurement.

## RESULTS

### APTASHAPE panel training

**Figure 1.**
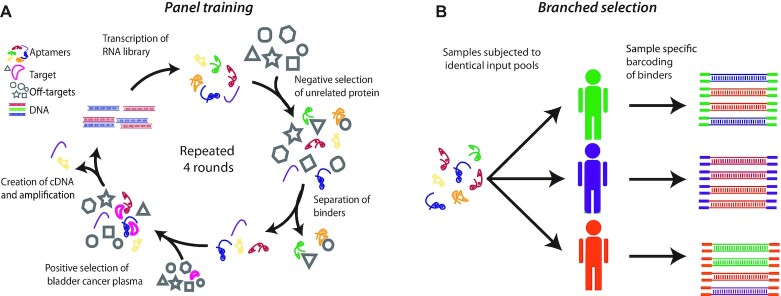
Workflow for APTASHAPE analysis. (**A**) A randomized RNA library is subjected to SELEX on the sample type of interest (referred to as panel training). RNA aptamers binding to plasma from control individuals are depleted from the library pool (negative selection). The remaining aptamers are exposed to a mixture of plasma from bladder cancer patients (positive selection) and actively binding aptamers are reverse transcribed, PCR-amplified, and transcribed back into RNA. This process is repeated four times to create a panel of aptamers enriched for their ability to bind biomolecules in the plasma of bladder cancer patients. (**B**) The trained panel of aptamers is used for selection against plasma from either control individuals (C, green), Ta stage- (purple) or T2–T4 cancer (orange) patients. Each sample type will produce a unique pattern of aptamer abundance, reflecting the composition of the sample.

The workflow in APTASHAPE is illustrated in Figure 1. A pool of 10^15^ 2′F-pyrimidine-modified RNAs containing a randomized region of 36 nucleotides was generated as a starting pool. The RNA molecules were incubated with total human plasma protein immobilized on magnetic beads and RNA aptamers with protein binding affinity were selected in an unbiased manner by performing four SELEX rounds (referred to as panel training; Figure 1A). For the panel training, plasma proteins pooled from five randomly selected patients diagnosed with bladder cancer stage T2–T4 (muscle-invasive) were used as bait. A negative selection step against plasma from control individuals was included in each SELEX round to enhance the representation of aptamers binding target proteins upregulated in plasma from cancer patients. The aptamer pools, enriched for binding to plasma protein from cancer patients, were sequenced after four and five rounds of panel training to evaluate the diversity of the RNA pool. In the RNA pool generated in round 4 (R4) the most abundant aptamers constitute about 10% of the sequences and >90% of aptamers represent individually <1% of the pool (Supplementary Figure S1). This pool was chosen as input for subsequent selection on individual patient samples.

### Applying APTASHAPE to plasma from bladder cancer patients

The R4 aptamer pool was subjected to one additional round of selection against plasma-derived proteins from individual bladder cancer patients (‘branched selection’; Figure 1B), hypothesizing that distinct shifts in the ratio of individual aptamer sequences between samples reflect alterations in the relative concentration of the proteins bound by that aptamer. A total of 96 plasma samples from a cohort comprising 32 controls without bladder cancer (C), 32 patients with bladder cancer stage Ta (non-muscle-invasive), and 32 patients with bladder cancer stage T2–T4 were included in the first study. The aptamer composition after each branched selection was determined by Illumina sequencing (generating an average of 3.5 million sequences per sample) and we focused on aptamers that change in relative abundance across the branched selection for individual patient samples. This led to recognition of 31 327 unique aptamers across all 96 samples tested. At the sequencing depth used, ∼4000 aptamers were detected in all samples at least 4 times (Supplementary Figure S2). Near identical sequences presumably originate from the same ancestor sequence and likely recognize the same protein. To reduce the data set, we then clustered the homologous sequences (Levenshtein–Damerau distance < 4), thereby reducing the number of aptamers to 13 258. To further reduce the burden of multiple hypothesis testing correction we continued our analysis for descriptive aptamers by selecting the 1000 most abundant sequences (averaged over all samples).

The counts and nucleotide sequence of the aptamers used for training and validation can be found in Supplementary Tables S2 and S3, respectively.

### Identifying aptamers descriptive of cancer and tumor progression stages

**Figure 2.**
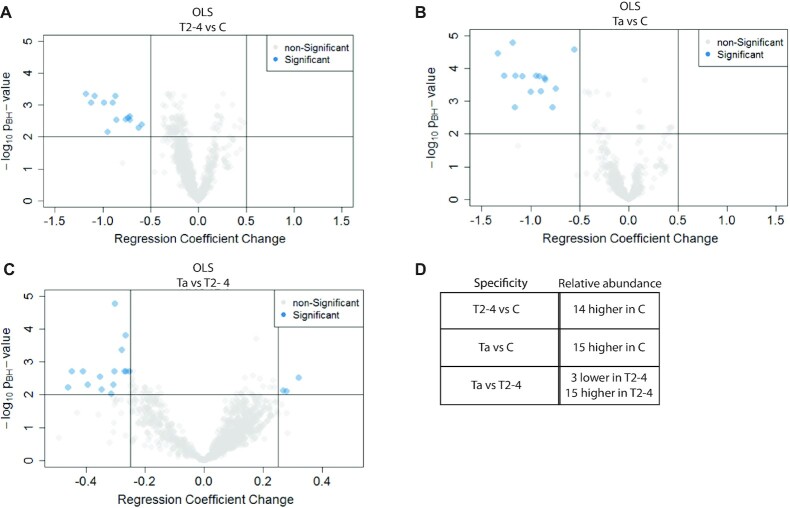
Volcano plots of *P*-values and fold changes in Ordinary Least Squares analysis of training set. The 1000 most abundant aptamers in the R4 pool, across all 96 branched selections, were tested for statistical significance when using OLS for each aptamer. A change in regression coefficient of 0.5 (for C (control) versus Ta (early stage cancer) or T2–T4 (late stage cancer)) or 0.25 (for Ta versus T2–T4) and a Benjamini–Hochberg adjusted *P*-value of 0.01 were defined as significant for all comparisons. (**A**) Comparison between T2–T4 and C showed 14 aptamer sequences to be significantly downregulated in T2–T4 samples. (**B**) Comparison between Ta and C showed 15 aptamer sequences to be significantly downregulated for Ta versus C; 14 of these are likewise specific for T2–T4 versus C. (**C**) Comparison between Ta and T2–T4 showed 18 aptamer sequences to be significantly different in abundance in Ta samples versus T2–T4. (**D**) Summary of the specificity of the descriptive aptamers.

For each of the 96 plasma samples analyzed, the abundance of the 1000 sequences was normalized to percent of total reads to enable comparison of aptamer levels between samples. Furthermore, aptamer levels were divided by the mean across all 96 samples to compare the degree of variation between individual aptamers. Next, Ordinary Least Squares regression analysis was applied to determine the ability of individual aptamers to distinguish plasma samples from patients with Ta and T2–T4 stage cancer from C using plasma samples from control patients as baseline. For identification of aptamers capable of discriminating between patients with Ta and T2–T4 tumors, Ta samples were used as baseline. *P*-values were calculated according to a Student's T-distribution using the Benjamini–Hochberg procedure for multiple testing correction. The average difference in aptamer level between sample types (C, Ta and T2–T4) as well as the *P*-values for each of the 1000 sequences were evaluated using volcano plots (Figure 2). Aptamers capable of differentiating between cancer stages were defined based on two criteria. They must 1: exhibit a significant shift in abundance ratio when comparing patient groups (>0.5 change in regression coefficient between Ta or T2–T4 samples and C, or >0.25 between Ta and T2–T4 samples), 2: have a Benjamini–Hochberg adjusted *P*-value <0.01.

According to these criteria, a total of 33 aptamers were deemed discriminatory of the disease conditions when analyzing plasma samples (Table 1). The aptamers were designated as depleted or increased in one type of samples relative to another based on the change in the regression coefficients. Exact values are shown in Supplementary Table S1. Fourteen aptamers were depleted in branched selections for plasma samples from patients diagnosed with bladder cancer versus C individuals, while one was specifically depleted in plasma from T2–T4 patients compared to C individuals (Figure 2A, B). The remaining 18 aptamers were all significantly different in abundance when analyzing plasma from Ta patients versus T2–T4 patients (Figure 2C). Of these, three were on average more abundant in branched selections on plasma from Ta patients than in T2–T4, while 15 exhibited higher average abundance in branched selections on plasma from T2–T4 patients than in Ta. These numbers are likewise summarized in Figure 2D. A search for common sequence motifs in the 33 aptamers revealed that 24 of them constitute nine families (named A–I) exhibiting distinct sequence motifs (Table 1), suggesting that each aptamer family may bind similar protein targets. The remaining nine aptamers did not exhibit any common extended sequence motifs.

**Table 1.** Descriptive aptamers identified using APTASHAPE

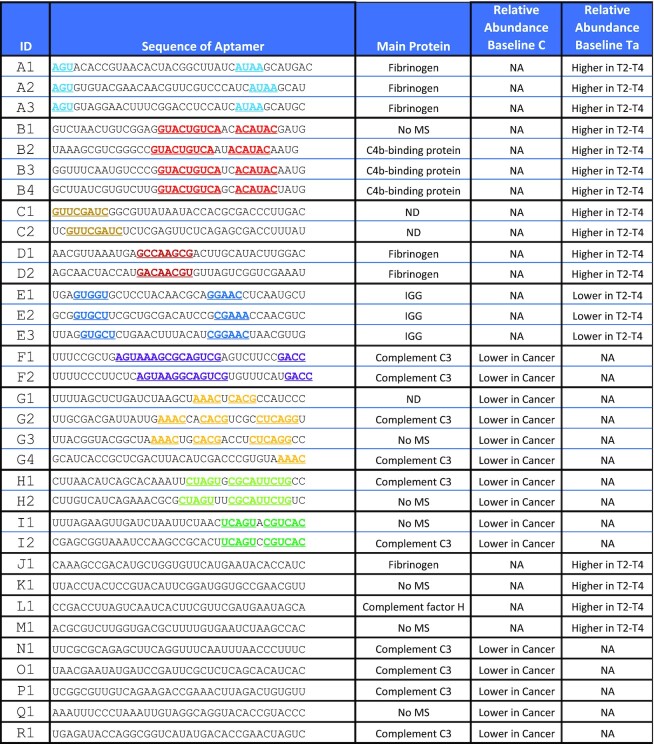

Sequence composition and enriched motifs for the 33 descriptive aptamers grouped by sequence similarity. The main proteins detected by mass spectrometry in the aptamer pull-down are noted in the third column. ND indicates that no significant protein was found. Aptamers that were not used for pull-down experiments are denoted ‘no MS’. The relative abundance relative to the C (Control) or Ta (early stage cancer) stages is noted in the fourth and fifth column, respectively, based on the analysis shown in Figure 2. Raw regression coefficients and *P*-values are shown in Supplementary Table S1.

### Validation of descriptive aptamers on a second patient cohort

**Figure 3.**
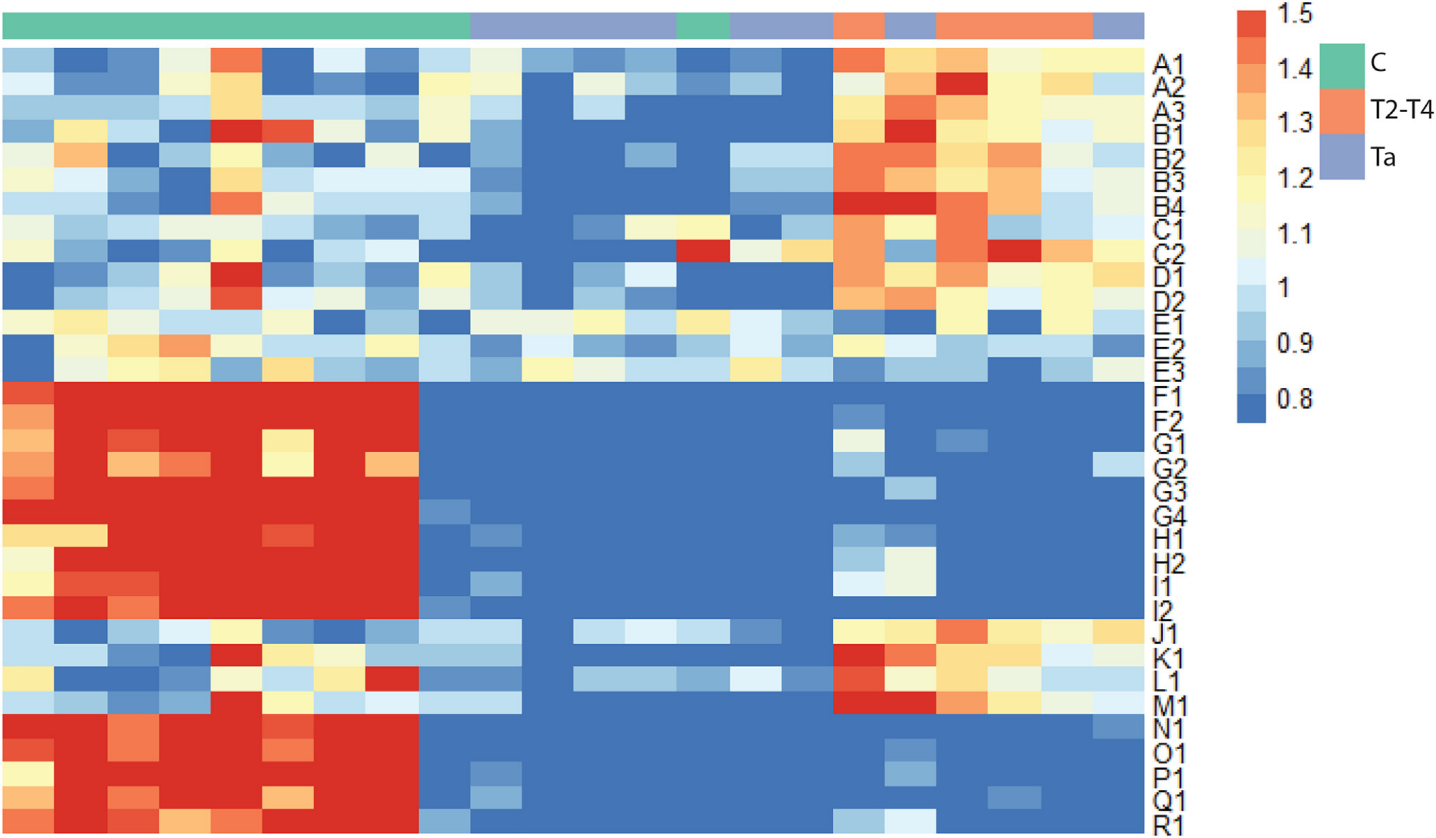
The descriptive aptamers separate disease stage in validation samples. The 33 discriminatory aptamers identified in the first selection round retain discriminatory power in a second patient cohort. The data shows the relative abundance for each aptamer compared to the mean across all samples. Columns were hierarchically clustered based on the binding profiles using complete linkage. Rows were arranged according to the sequence families shown in Table 1. Red color in the heatmap shows high relative abundance, white shows average relative abundance and blue shows low relative abundance.

**Figure 4.**
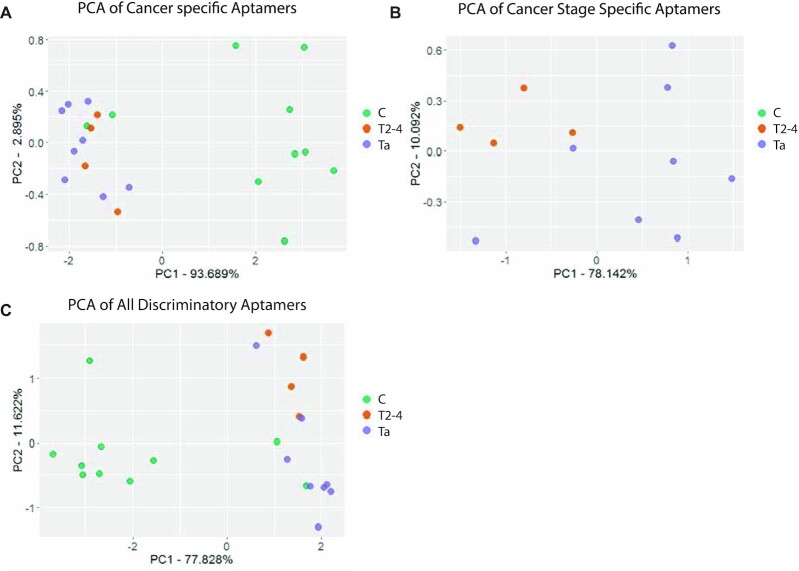
PCA plots of validation samples using discriminatory aptamers. Principal component analysis of the validation samples using the aptamers found to be discriminatory for either cancer stage vs control (**A**) or Ta versus T2–T4 (**B**). (**C**) Combined PCA with the discriminatory aptamers from (A) and (B). Here, the first component mainly separates the C samples from the cancer samples and the second component mainly separates the Ta stage from the T2–T4 samples.

To validate the ability of the 33 aptamers to differentiate between plasma samples from patients with various stages of bladder cancer and controls, we performed branched selection on plasma samples from an independent bladder cancer cohort containing eight Ta, four T2–T4, and 10 Cs using the same R4 aptamer pool as input. The aptamer composition after branched selection was determined by Illumina sequencing as described above, yielding an average of 4.4 million reads per plasma sample. We found all 33 discriminatory aptamers from the 1st cohort to be present in the branched selections for the second validation cohort samples and performed an unsupervised hierarchical clustering of the samples based on changes in aptamer ratios (Figure 3). The heatmap shows that the 33 discriminatory aptamers have a similar pattern of differential enrichment and depletion in the validation cohort as compared to 1st cohort. The descriptive aptamers fall into the same two groups as for the first patient cohort: 15 aptamers could discriminate between plasma from cancer patients (Ta + T2–T4) and controls (with two individuals possibly miscategorized) and 18 aptamers could distinguish between plasma from Ta and T2–T4 patients, the behavior of each aptamer in the heatmap was consistent with the predicted behavior from the OLS (Table 1). The ability of the 33 discriminatory aptamers to distinguish between high and low cancer stage in the second cohort was further investigated by principal component analysis (PCA; Figure 4). In Figure 4A, all 2nd cohort samples were plotted according to the levels of the 15 sequences descriptive of Ta or T2–T4 versus controls. This showed that cancer status of the patient is the factor that best explains the variation in the data set (PC1, 94% variation explained) when comparing plasma samples from controls and cancer patients.

We performed a separate principal component analysis for the Ta and T2–T4 plasma samples from the 2nd cohort using the 18 aptamers descriptive of cancer stage and saw a clear but less pronounced clustering of the samples (Figure 4B). The control samples were separated from the cancer samples on principal component 1 (78% variation), while the Ta and T2–T4 samples were separated on principal component 2 (10% variation). This shows that the main variation described by the cancer stage-descriptive aptamers in the second cohort is between sample types. In Figure 4C, all second cohort samples were plotted according to the levels of the 33 discriminatory aptamers. This showed that cancer status (disease versus control) aligns well with the major contributor to data variation for this sequence data set (PC1, 78% variation explained) and that cancer stage aligns with the second largest contributor to data variation (PC2, 12% variation explained). Based on the coordinates in Figure 4C it is possible to arrange the samples into groups. Healthy and cancer status is determined by the first component, with a positive coordinate indicating cancer and a negative coordinate indicating that the plasma sample is from a control. Using this, we calculated the accuracy of differentiating control vs cancer samples to be 91%. For the same dataset, the early and late stage cancers can be separated on the second component, with the late stage showing positive coordinates and the early stage having negative coordinates. Using this, we calculated the accuracy for early stage versus late stage to be 83%. We conclude that the descriptive power of the aptamers identified using the first patient cohort could be validated in a second patient cohort.

### Identification of proteins bound by the discriminatory aptamers

We hypothesized that the differential abundance of aptamers after branched selection mirrors the differential expression or modification of target proteins in the patient plasma samples, and that the aptamers provide a means to affinity-purify these proteins. To identify the protein targets, 26 of the 33 cancer-discriminatory aptamer were generated individually by *in vitro* transcription, tagged with biotin for capture onto streptavidin magnetic beads and incubated with plasma. After extensive washing, the aptamer-bound proteins were analyzed by SDS-PAGE and the most prominent bands were excised and subjected to mass spectrometry (Supplementary Figure S3A–I). Similarities in protein patterns between aptamer pull-downs on the SDS-PAGE gels suggested that some of the discriminatory aptamers may bind the same protein; this was particularly frequent for aptamers that share common sequence motifs. Notably, most of the aptamers pulled down a mixture of proteins, which suggests that the proteins recognized by the aptamers are part of larger complexes. As a control, we included a pull-down using empty beads (Supplementary Figure S3I). The protein bands appearing in this sample were disregarded across all aptamer pull-downs, as they likely reflect non-specific binding to highly abundant plasma proteins.

Mass spectrometry (MS) analysis of the excised bands was used to identify the proteins bound by each aptamer (Table 1). The target proteins bound by the aptamers were defined as the most abundant protein fragments in the MS with a score of at least 500 and at least twice the score of the highest score of keratin, a common contaminant in MS data derived from plasma samples. The 26 aptamers tested bound five main proteins: Complement C3, Fibrinogen (FBG), C4b-binding protein (C4BP), Complement factor H (CFH) and Immunoglobulin gamma (IgG). In addition to these main proteins, several other proteins including Protein S (PROS1), Apolipoprotein E (APOE) and Haptoglobin (HP) were found associated with the aptamers. Albumin (ALB) was present in most pull-downs, including the negative control with no aptamers, and therefore reflects non-specific binding (Supplementary Figure S3I, lane NA). As expected, aptamers within the same family (based on shared sequence motifs) were found to bind the same protein (Table 1), supporting the observation from SDS-PAGE that several aptamers with closely related sequence motifs share the target protein.

## DISCUSSION

Currently, there is an unmet need for high throughput technologies to screen the protein content in biological samples. We have developed an RNA aptamer-based protein profiling tool, APTASHAPE, for patient sample analysis and potential biomarker discovery. We demonstrate that dynamic changes in the aptamer pools after branched selection on plasma from bladder cancer patients can be used to discriminate between samples from cancer patients and healthy individuals, and it can be used to discriminate between cancer stages. Presumably, the changes in aptamer profiles seen among the branched selections reflect differences in the global proteome of the plasma samples (e.g. protein concentration, post-translational modifications or formation of larger complexes). Differential selection of aptamers to biological samples has been reported before. Domenyuk *et al.* showed that DNA aptamer pools generated by tissue-SELEX on formalin-fixed paraffin-embedded (FFPE) tumor samples could distinguish trastuzumab-responding breast cancer patients from non-responders in combination with chemotherapy (24). In another study, aptamer panels raised against exosomes allowed stratification of samples from healthy, breast cancer biopsy-negative, and -positive women (9). Also, a pilot study performed on blood serum from 16 mice (10 transgenic mice that mimic Alzheimer's disease, 6 healthy mice) reported that a combination of DNA aptamers could distinguish between the different types of mice; however this was not validated in an independent cohort and no targets were identified (25).

In the present study, plasma samples from two cohorts of bladder cancer patients and controls were profiled using chemically modified RNA aptamers, one for developing a set of diagnostic aptamers and one for validating the predictive power of these aptamers on a second set of samples. This cross-validation confirmed the robustness of the APTASHAPE method and enabled us to provide a set of specific aptamers that can distinguish between plasma samples from bladder cancer patients and controls with high level of confidence and even, with a significant level of accuracy, discriminate between non-invasive and invasive bladder cancer stages. Importantly, APTASHAPE enables discrimination of the disease status independently of target identification. However, to better understand the basis for the Aptashape result, an MS analysis was performed and it showed that the discriminatory aptamers bind at least five different main proteins, several of which are factors in the complement system.

The protein bound by the highest number of discriminative aptamers (ten) is Complement factor C3 (C3), a central factor in both the classical and alternative complement system (26). Upon activation, C3 is cleaved and deposited on pathogenic cells (27), which means that an active infection or pathogenic condition is expected to reduce C3 levels in the blood as shown for bladder cancer patients (28). In agreement with this, we found that all C3-binding aptamers had a higher relative abundance in control samples than cancer samples. The SDS-PAGE for pull-downs using C3 binding aptamers showed lower molecular weight bands, which may be C3 cleavage products. In addition, the MS data for several of the C3-binding aptamers also revealed the presence of Complement factor H, a known inhibitor of C3 activation (Supplementary Figure S3; see below).

Fibrinogen was recognized by the second-highest number of aptamers (six). Fibrinogen is an important part of the coagulation system (29) but it is also involved in inflammation and apoptosis (30) and a previous study showed that increased fibrinogen levels in blood correlated with a poor prognosis in bladder cancer (31). Consistent with this, the fibrinogen-binding aptamers were all enriched in branched selections of plasma from late-stage cancer patients.

C4b-binding protein (C4BP) is recognized by three aptamers. It is a large multimer component of the complement system that binds activated C4b and promotes its degradation (32). C4BP is known to bind Protein S (PROS1), which may help to localize complement regulatory activity to certain cells (33). Three C4BP-binding aptamers also pulled down Protein S and these aptamers showed increased abundance in branched selections from late-stage bladder cancer patients. This observation agrees with previous reports that C4BP levels are elevated in some cancers (34,35) and that Protein S is related to poor prognosis in bladder cancer (36, https://www.proteinatlas.org/ENSG00000184500-PROS1/pathology/urothelial+cancer).

Immunoglobulin gamma (IgG) accounts for 10–20% of plasma proteins and is an important part of the humoral immune response. Multiple subtypes of IgG are found in the body with differing specificities and functions (37). IgG is recognized by three discriminatory aptamers, which all show increased rates in plasma from Ta patients compared to T2–T4 patients. It has been shown in the literature that tumor tissue in bladder cancer patients expresses IgGs, which are distinct from normal B-cell derived IgGs (38) but the MS data obtained did not enable us to determine which type of IgG these aptamers bind.

A single aptamer bound Complement factor H (CH) as the main protein. CH is another complement component, known to bind C3 and inhibit its activation (26,27). We found CH to be present in small amounts in most of the pull-downs for C3-binding aptamers, consistent with C3–CH complex formation for a subpopulation of the aptamer-bound C3. Interestingly, the major CH binding aptamer (L1) lacks the C3 band (lane L1 in Supplementary Figure S3G), suggesting that it preferentially binds the CH monomer. The CH monomer-specific aptamer is enriched in T2–T4 samples, in contrast to the C3-binding aptamers that have a higher relative abundance in the control samples. This opposite behavior of aptamers binding C3–CH and CH only is consistent with a report that found higher levels of complement factor H in urine of bladder cancer patients (38).

Altogether, five main proteins identified by MS have previously been found to show altered expression in bladder cancer, supporting the ability of APTASHAPE to identify proteins with a putative biomarker function. Notably, even large complexes such as C4BP-Protein S were identified by MS of affinity-purified proteins, underlining the ability of APTASHAPE to target naturally occurring protein complexes rather than individual proteins. We previously showed that aptamers are able to distinguish between different post-translationally modified forms of a single protein (12), and it is likely that some of the changes in aptamer composition observed here reflect the presence of multiple protein isoforms. It remains to be studied how much these variables influence our APTASHAPE results.

Although the signals obtained from mass spectrometry and APTASHAPE both are affected by the abundance of particular biomarker proteins there are also fundamental differences in their mechanism of surveillance. Where mass spectrometry directly measures the abundance of the proteins (or peptides) the APTASHAPE readout is reflecting the exposure or disappearance of epitopes as a result of conformational changes and complex formation in plasma protein.

Using aptamer-based pull-downs for validation of potential biomarkers by mass spectrometry strongly reduces background noise compared to mass spectrometry of plasma samples from different patients. Considering the thousands of proteins present in plasma, we see only a few cases of unspecific protein binding, i.e. proteins pulled down irrespective of the aptamer used, in the aptamer pull-downs (Supplementary Figure S3). Applying mass spectrometry analysis of the affinity purified samples will lower patient-to-patient variation as well as the burden of multiple hypothesis testing is reduced, enabling more significant predictions. The disadvantage of aptamer-based biomarkers is that only proteins that are recognized by a specific aptamer in the selected pools contribute to the prediction algorithm. For computational reasons, only the 1000 most abundant aptamer sequences from the branched selections were used in our analysis. This may explain why the main protein targets found were relatively abundant blood proteins, including C3, which was bound by 13 individual aptamers. Of these, 10 aptamers belong to four different sequence families while three represent unique sequences. Previous studies of aptamers targeting complex mixtures also showed that a few specific aptamers often dominate the selection (39–41). Detection of more lowly concentrated proteins may require a deeper sequencing of the branched selection samples.

In our selection, the target protein with the lowest reported concentration in blood (CH, 3 μM) (42) is only recognized by a single aptamer. Increasing the number of aptamers included in the training of the computational model would most likely allow us to detect less abundant target proteins. However, the decrease in signal to noise ratio due to Poisson noise poses a challenge for analyzing low-abundant aptamers. This can be solved either by actively depleting the most abundant sequences from the pool at an experimental level, or by increasing the total amount of reads from the sequencing to cover rare aptamers with more reads. It is also possible that the aptamers for which no targets could be identified recognize proteins that are not sufficiently abundant in plasma to be detectable by MS.

In this study, the SELEX procedure is performed using untreated human plasma, unlike previous studies that used purified exosomes (9) or tissue slices (11). Only 1 μl of plasma is sufficient for running a branched selection, which complies with minimal invasiveness for the patient and which opens the possibility to develop bedside diagnostic assays with a pool of defined aptamers. In addition, the low production costs and stability during storage make aptamers prime candidates for non-invasive point-of-care detection. Although the work here was done using plasma, which requires an additional processing step before point-of-care detection compared to whole blood, APTASHAPE may be expanded to work on other unprocessed biofluids, such as blood or spinal fluid, as long as the library training is performed on the same biofluid. As opposed to aptamers selected to purified targets under non-physiological conditions, the individual aptamers selected by APTASHAPE function optimally in biofluids and can therefore be applicable to other types of detection devices and eventually as drug candidate. The APTASHAPE aptamers are also likely to work well in combination as they were originally selected under combinatorial conditions.

We conclude that APTASHAPE can differentiate plasma samples from bladder cancer patients and healthy controls with high level of accuracy and even, to some extent, distinguish between cancer stages. Finally, APTASHAPE provides a discovery tool for potential biomarker proteins in plasma that may represent targets for future therapy.

## DATA AVAILABILITY

The sequences used for the training and validation are found in the Supplementary Table S2.

The mass spectrometry proteomics data have been deposited to the ProteomeXchange Consortium via the PRIDE partner repository with the dataset identifier PXD032972. Scripts available on: https://github.com/SorenFjel/.

## Supplementary Material

zcac025_Supplemental_Files
