# Supplementary material for: Differential RNA aptamer affinity profiling on plasma as a potential diagnostic tool for bladder cancer
Source: NAR Cancer. 2022 Aug 22;4(3):zcac025. doi: 10.1093/narcan/zcac025 (PMC9394167; doi:10.1093/narcan/zcac025)
Supplement: zcac025_Supplemental_Files [file zcac025_supplemental_files.zip › Supplementary_Data.pdf]

Supplementary information

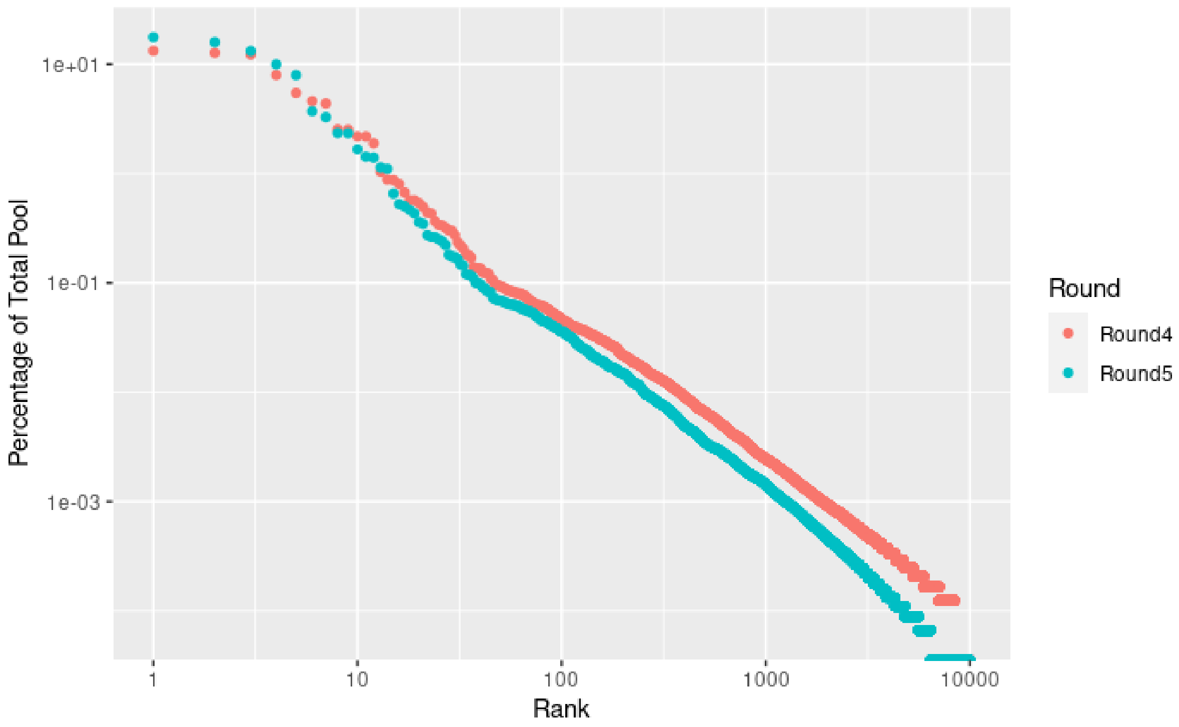

Supplementary Figure S1: Aptamer sequences ranked according to number of reads in pools after 4th and 5th round of selection.

RNA pools were sequenced after 4 and 5 rounds of panel training, respectively. The sequence read counts were converted to percentage of total pool. The 10.000 most abundant sequences were sorted by abundance and plotted in this graph.

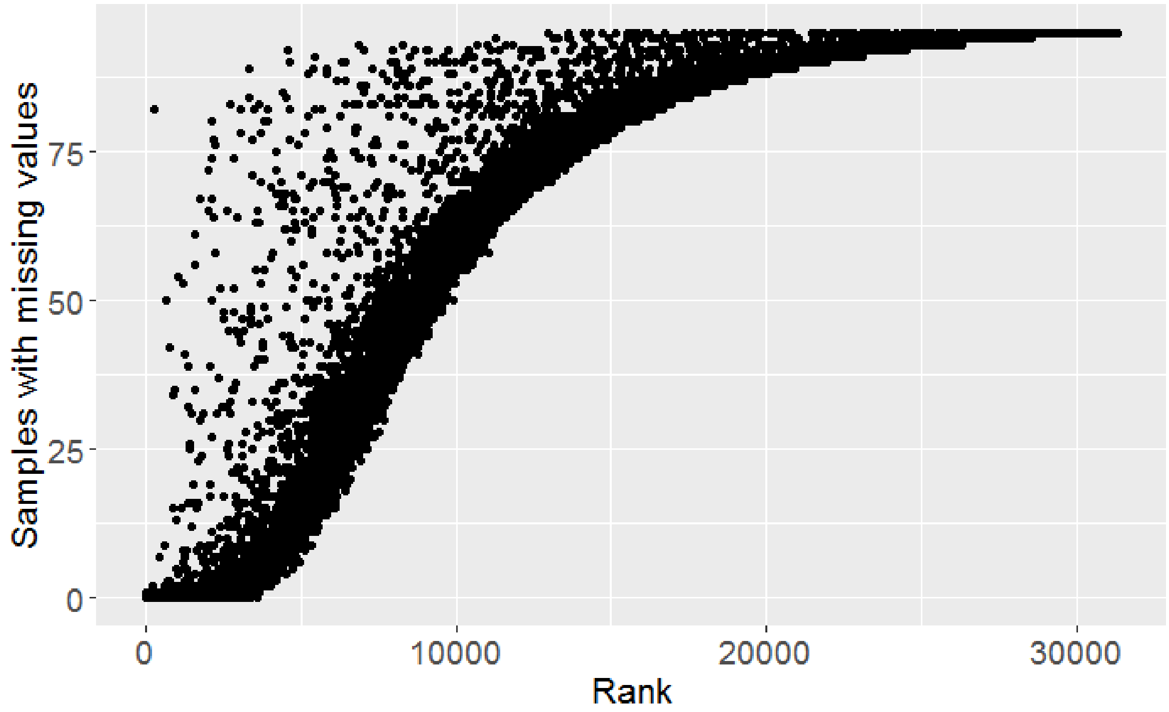

Supplementary Figure S2: Amount of samples with <4 reads (y-axis) plotted against sequences ranked according to the abundance across all samples (x-axis).

ID	Sequence of Aptamer	Change in Regression coefficient C vs Ta	-log10 pvalue Control vs Ta	Change in Regression coefficient Ta vs T2-T4	-log10 pvalue Ta vs T2-T4
A1	AGUACACCGUAACACUACGGCUUAUCAUAAGCAUGAC	-0.24	1.02	-0.39	2.29
A2	AGUGUGUACGAACAACGUUCGUCCCAUCAUAAGCAU	-0.28	0.92	-0.46	2.22
A3	AGUGUAGGAACUUUCGGACCUCCAUCAUAAGCAUGC	-0.26	1.73	-0.31	2.01
B1	GUCUAACUGUCGGAGGUACUGUCAACACAUACGAUG	-0.11	0.68	-0.26	3.79
B2	UAAAGCGUCGGGCCGUACUGUCAAUACAUACAAUG	-0.12	0.41	-0.30	2.71
B3	GGUUUCAAUGUCCCGGUACUGUCAUCACAUACAAUG	-0.08	0.27	-0.25	2.71
B4	GCUUAUCGUGUCUUGGUACUGUCAGCACAUACUAUG	-0.04	0.14	-0.27	2.71
C1	GUUCGAUCGGCGUUAUAAUACCACGCGACCCUUGAC	-0.21	1.91	-0.26	2.71
C2	UCGUUCGAUCUCUCGAGUUCUCAGAGCGACCUUUAU	-0.35	2.27	-0.35	2.16
D1	AACGUUAAAUGAGCCAAGCGACUUGCAUACUUGGAC	-0.17	0.48	-0.45	2.71
D2	AGCAACUACCAUGACAACGUGUUAGUCGGUCGAAAU	-0.17	0.81	-0.35	2.55
E1	UGAGUGGUGCUCCUACAACGCAGGAACCUCAAUGCU	-0.07	0.16	0.32	2.55
E2	GCGGUGCUUCGCUGCGACAUCCGCGAAACCAACGUC	0.18	0.79	0.28	2.12
E3	UUAGGUGCUCUGAACUUUACAUCGGAACUAACGUUG	-0.05	0.11	0.27	2.12
F1	UUUCCGCUGAGUAAAGCGCAGUCGAGUCUUCCGACC	-1.18	4.78	-0.43	1.45
F2	UUUUCCCUUCUCAGUAAGGCAGUCGUGUUUCAUGACC	-1.08	3.75	-0.15	0.35
G1	UUUUAGCUCUGAUCUAAGCUAAACUCACGCCAUCCC	-0.86	3.71	-0.31	1.14
G2	UUGCGACGAUUAUUGAAACCACACGUCGCCUCAGGU	-0.74	3.38	-0.19	0.83
G3	UUACGGUACGGCUAAAACUGCACGACCUCUCAGGCC	-1.33	4.46	-0.27	0.75
G4	GCAUCACCGCUCGACUUACAUCGACCCGUGUAAAAC	-1.27	3.77	-0.24	0.55
H1	CUUAACAUCAGCACAAAUUCUAGUGCGCAUUCUGCC	-0.77	2.80	-0.05	0.11
H2	CUUGUCAUCAGAAACGCGCUAGUUUCGCAUUCUGUC	-0.89	3.29	-0.18	0.40
I1	UUUAGAAGUUGAUCUAAUUCUAACUCAGUACGUCAC	-0.94	3.77	-0.31	0.87
I2	CGAGCGGUAAAUCCAAGCCGCACUUCAGUCCGUCAC	-0.91	3.75	-0.06	0.18
J1	CAAAGCCGACAUGCUGGUGUUCAUGAAUACACCAUC	-0.25	1.43	-0.41	2.71
K1	UUACCUACUCCGUACAUUCGGAUGGUGCCGAACGUU	-0.11	0.78	-0.30	4.76
L1	CCGACCUUAGUCAAUCACUUCGUUCGAUGAAUAGCA	-0.12	0.33	-0.31	2.29
M1	ACGCGUCUUGGUGACGCUUUUGUGAAUCUAAGCCAC	-0.05	0.15	-0.28	3.37
N1	UUCGCGCAGAGCUUCAGGUUUCAAUUUAACCCUUUC	-1.16	2.80	-0.32	0.54
O1	UAACGAAUAUGAUCCGAUUCGCUCUCAGCACAUCAC	-0.85	3.67	-0.19	0.62
P1	UCGGCGUUGUCAGAAGACCGAAACUUAGACUGUGUU	-1.16	3.77	-0.11	0.19
Q1	AAAUUUCCCUAAAUUGUAGGCAGGUACACCGUACCC	-1.00	3.27	-0.20	0.45
R1	UGAGAUACCAGGCGGUCAUAUGACACCGAACUAGUC	-0.55	4.56	-0.22	1.44

Supplementary Table S1: Values from OLS analysis of aptamers

The change in regression coefficient and negative log10 of the individual aptamers from the OLS analysis shown in Figure 2. The least progressed sample is used as baseline in all cases, i.e. the control group C is used as baseline vs Ta, and Ta is used as baseline against T2-T4.

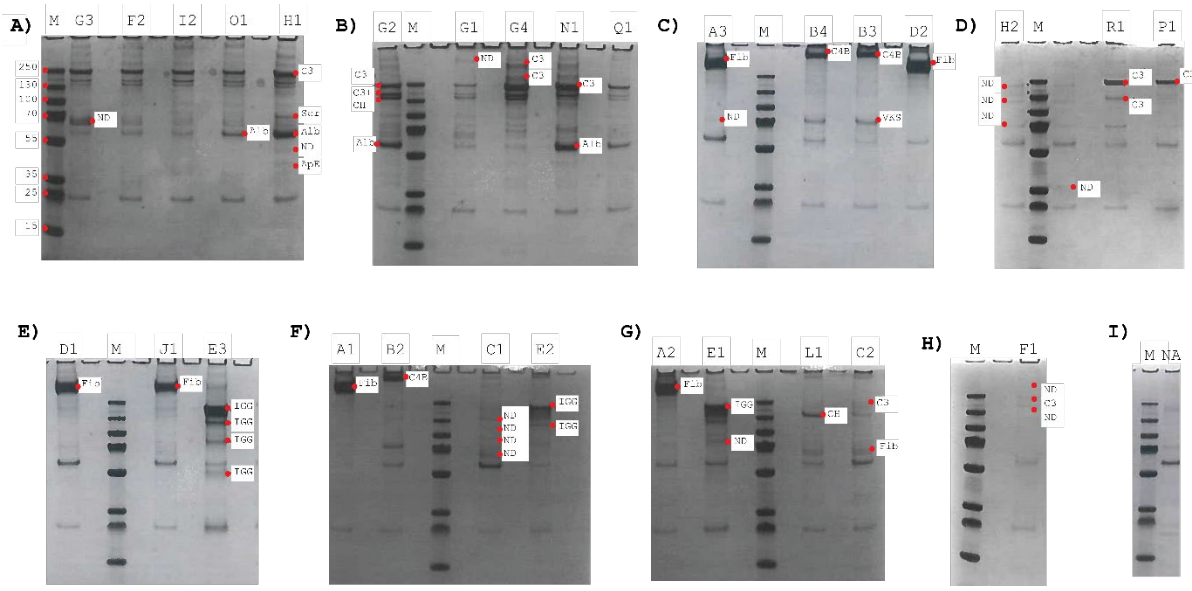

Supplementary Figure S3: Coomassie Blue stained SDS-PAGE-gels of protein obtained by aptamer pulldown

Affinity purification of plasma proteins using immobilized aptamers. Eluted proteins were fractionated by SDS-PAGE and the indicated bands were excised and subjected to mass spectrometry. The aptamer names are listed above each lane, M indicates the protein marker (see panel A for sizes). The labels indicate the identity of proteins with a signal at least 2 times stronger than keratin in the sample. ND: Non Determined, Alb: Albumin, C3: Complement C3, Ser: Serotransferrin, CH: Complement factor H, Fib: Fibrinogen, C4B: C4b-binding protein, VKS: Vitamin K-dependent protein S, IGG: Immunoglobulins. NA: No aptamer used, only beads.
